# *Agrimonia pilosa* Ledeb. aqueous extract improves impaired glucose tolerance in high-fat diet-fed rats by decreasing the inflammatory response

**DOI:** 10.1186/s12906-017-1949-z

**Published:** 2017-09-05

**Authors:** Hwan Hee Jang, Song Yee Nam, Mi Ju Kim, Jung Bong Kim, Jeong Sook Choi, Haeng Ran Kim, Young Min Lee

**Affiliations:** 1 Functional Food & Nutrition Division, National Institute of Agricultural Sciences, Rural Development Administration, Wanju, 55365 Republic of Korea; 2 Division of Applied Food System, Major of Food and Nutrition, Seoul Women’s University, Seoul, 01797 South Korea

**Keywords:** *Agrimonia Pilosa*, Glucose tolerance, High-fat diet, Inflammation, Obesity

## Abstract

**Background:**

*Agrimonia pilosa* Ledeb. is a medicinal plant with physiological activities such as anti-cancer, antioxidant, anti-inflammatory activities and in vitro anti-diabetic activity. However, the effects of aqueous extracts from *A. pilosa* on insulin-resistant rats have not yet been examined. We investigated the effects of aqueous extract from *A. pilosa* on impaired glucose metabolism induced by a high-fat diet in rats.

**Methods:**

Male Sprague-Dawley rats were assigned to the following groups: normal-fat diet (NF, *n* = 9); high-fat diet (HF, *n* = 9); high-fat diet with 0.1% *A. pilosa* aqueous extract (HFA, *n* = 10). Experimental diets were administered for 16 weeks. At the end of the treatment, liver and fat tissues were isolated, and serum was collected for biochemical analysis.

**Results:**

The HF group rats had a significantly higher liver weight than the NF group rats did, and increased hepatic lipid accumulation (*p* < 0.05); however, supplementation with *A. pilosa* decreased liver weight. Blood glucose levels in the HFA group were lower than levels measured in the HF group 30, 60, and 120 min after glucose administration (*p* < 0.05). In addition, dietary *A. pilosa* supplementation decreased tumor necrosis factor α and interleukin 6 levels, while increasing serum adiponectin concentrations (*p* < 0.05 vs. the HF group). These effects were accompanied by reduced hepatic and adipose tissue expression of inflammation-related genes such as *Tnf* and *Il1b* (*p* < 0.05).

**Conclusions:**

Our findings indicate that *A. pilosa* aqueous extract can ameliorate insulin resistance in high-fat diet-fed rats by decreasing the inflammatory response.

**Electronic supplementary material:**

The online version of this article (10.1186/s12906-017-1949-z) contains supplementary material, which is available to authorized users.

## Background

The incidence of obesity has risen dramatically during the past 50 years. In Korea, obesity (defined as having a body mass index greater than 25 kg/m^2^) is prevalent in 32.9% of the adult population [1]. Obesity affects adults and children worldwide, and its recent dramatic increase has been caused by decreased energy expenditure and increased energy intake.

Excessive calorie intake usually causes obesity, glucose intolerance, and insulin resistance [2]. Insulin resistance is defined as a decreased sensitivity of the peripheral tissues to insulin action [3]; it increases the risk of cardiovascular disease, type 2 diabetes, hypertension, and nonalcoholic fatty liver disease (NAFLD) [4]. Insulin resistance is also known to cause a metabolic syndrome, which manifests as steatosis in hepatic tissue [5].

NAFLD has been defined as the accumulation of hepatic lipid not due to excess alcohol consumption. It encompasses a spectrum of disorders, ranging from hepatic steatosis to nonalcoholic steatohepatitis (NASH), cirrhosis, and hepatocellular carcinoma [6]. Although NAFLD per se may be a benign process, it can progress to inflammatory liver disease. Its high prevalence and contribution to causes of death are also an important issue for management of life expectancy [7]. Therefore, NAFLD should be diagnosed and regulated early, before it progresses.

It is now well-established that insulin resistance-related metabolic syndrome and NAFLD are associated with a chronic inflammatory response, also termed metaflammation [8]. Chronic low-grade inflammation is characterised by the increased production of inflammatory adipose tissue-specific secretory proteins (adipokines), such as tumor necrosis factor (TNF) α and interleukin (IL) 6, contributing to glucose intolerance and insulin resistance. Unlike levels of other adipokines, those of adiponectin (also known as Acrp30, AdipoQ, and GBP28) decrease in obesity-induced metabolic disorders, including insulin resistance [9].

*Agrimonia pilosa* Ledeb. is a medicinal plant known to exert anti-cancer [10] and antioxidant [11] effects, acetylcholinesterase inhibition [12], and anti-inflammatory activities [13, 14]. Our previous study indicated that *A. pilosa* extracts improved the free fatty acid-induced insulin resistance in C2C12 myotubes [15]. However, the effects of aqueous extracts from *A. pilosa* on insulin-resistant rats have not yet been examined. The purpose of this study was to investigate the effects of *A. pilosa* aqueous extract on insulin resistance induced by a high-fat diet (HFD) in rats.

We used HFD to establish glucose intolerance, and investigated the effects of *A. pilosa* treatment on glucose tolerance and the potential underlying molecular mechanisms.

## Methods

### Plant material and extraction

The aerial parts of *A. pilosa* were purchased from the Gokseong Agricultural Association (Jeollanam-do, Korea) in 2011 as dried form and identified by the Classification and Identification Committee of the Korea Institute of Oriental Medicine (KIOM). A voucher specimen (KIOM109-122Aa) was kept in the herbarium of the Department of Herbal Resources Research of the KIOM. Dried *A. pilosa* was extracted twice with 10 volumes of water at 80 °C for 3 h. The extracts were filtered through No. 6 filter paper (Whatman, Maidstone, UK) and were concentrated under reduced pressure by a rotary evaporator (EYELA, Tokyo, Japan) at 40 °C. The water filtrates were frozen and lyophilised (Ilshin Lab). The final lyophilised extracts were stored at − 20 °C until use.

### Animals and diets

Seven-week-old male Sprague-Dawley (SD) rats with an average weight of 271.9 ± 8.7 g were purchased from Orient Bio Co. Ltd. (Seongnam, Korea). All rats were housed individually in plexiglass cages and maintained in an air-conditioned room at 22 ± 2 °C under an automatic lighting schedule. To help with adaptation to the laboratory conditions, the rats were fed a standard pellet diet with free water for the first week prior to the experiment. After the acclimation period, the rats were randomly blocked into three groups (*n* = 10 per group) with similar mean body weights. The rats were assigned to the following groups: Normal-fat diet (NF); HFD (HF, 36% of energy as fat); HFD with 0.1% *A. pilosa* aqueous extract (HFA). These experimental diets were maintained for 16 weeks. The composition of the experimental diets is shown in Additional file 1. This experimental design was approved by the Institutional Animal Care and Use Committee (IACUC) of the National Academy of Agricultural Science (reference number: NAAS-1203).

### Preparation of blood and tissue samples

On the last day of the experiment, after fasting for 16 h, all rats were anesthetized with CO_2_ and blood was collected via the heart. The collected blood was kept in cold water for approximately 30 min, and the serum was separated by centrifugation at 3000 rpm for 20 min at 4 °C. After cutting the abdomen open, small pieces of liver and fat tissues were rapidly excised and frozen immediately in liquid nitrogen for Real-time quantitative reverse transcription PCR. The liver and fat tissues were removed, rinsed with cold phosphate-buffered saline, and weighed. Hepatic sections for histological evaluation were stored in 10% buffered neutral formalin. The residue was frozen immediately in liquid nitrogen and stored –in a − 70 °C freezer.

### Oral glucose tolerance test (OGTT)

Rats were subjected to an overnight fast for 16 h before the OGTT. The baseline glucose level was measured by OneTouch Ultra Glucose Monitoring System (Johnson & Johnson Medical, New Brunswick, NJ, USA). The 75% glucose solution (2 g/kg b.w.) was orally injected, and serum glucose in the blood sample from the tail vein was estimated by a glucometer at 0, 30, 60, and 120 min. Assays were carried out in triplicate (*n* = 10). The area under the curve (AUC) was also calculated.

### Blood biochemical assays

Serum glucose was determined using commercial assay kits (Asan Pharmaceutical, Seoul, Korea) according to the manufacturer’s protocols. Insulin and adiponectin levels were analysed by commercial assay kits (Millipore Corp., Billerica, MA, USA). Serum TNF-α and IL-6 levels were also measured using commercial assay kits (R&D Systems, Minneapolis, MN, USA) according to the manufacturer’s protocols. Assays were carried out in duplicate (*n* = 10). The homeostasis model assessment of insulin resistance (HOMA-IR) was obtained by the calculation (HOMA-IR = glucose (mg/dL) × insulin (mU/L)/405).

### Liver histology

Liver tissue was fixed with 10% neutral buffered formalin and embedded in paraffin. Sections were cut and stained with Oil red O staining. Images were captured using an Olympus AX 70 camera (Center Valley, PA, USA). The initial assessment was performed under low magnification (40 × to 200 ×), and confirmed under high magnification (400 ×). Hepatic steatosis was graded as 0 (fatty hepatocytes occupying < 5%), 1 (fatty hepatocytes occupying 5–33%), 2 (fatty hepatocytes occupying 34–66%), or 3 (fatty hepatocytes occupying > 66%) according to the percentage of hepatic lipid [16].

### Real-time quantitative reverse transcription PCR

Total RNA was isolated from liver and fat tissues using the RNeasy Microarray Tissue Kit (Qiagen, Valencia, CA, USA), and RNA integrity (RIN > 9.0) was assessed using a Bioanalyzer 2100 (Agilent Technologies, Santa Clara, CA, USA). The Rat Fatty Liver PCR array and Insulin Resistance PCR array (SABiosciences, Frederick, MD, USA) were used to profile the genes differentially expressed in liver and fat tissue, respectively, according to the manufacturer’s instructions. The complete list of genes assayed on the array is provided on the manufacturer’s website (http://www.sabiosciences.com/Metabolic_Diseases.php). For each plate, 0.5 μg of RNA was converted to double-stranded cDNA using the RT^2^ first strand synthesis kit (Qiagen). After mixing this with the SABiosciences RT^2^ qPCR master mix, the cDNA was pipetted into the 96-well profile plate and amplified on CFX96TM Real-Time PCR Detection System Bio-Rad Laboratories (Hercules, CA, USA). Data were normalised using lactate dehydrogenase A as an endogenous control, and fold-changes in expression were calculated using SABiosciences online software (http://pcrdataanalysis.sabiosciences.com/pcr/arrayanalysis.php).

### Statistical analysis

Data were expressed as mean ± standard error (S.E.). Statistical comparisons were performed by one-way ANOVA followed by Duncan’s multiple range test. Values were considered statistically significant when *p* < 0.05.

## Results

### Effects on body weight, food intake, and organ weight

Body weight, food intake, and food efficiency ratio (FER) are presented in Table 1. The experimental groups were rearranged by initial body weight (between 269.0 g and 274.6 g) before HFD feeding began. After 16 weeks on the assigned diets, the weight of rats in the HFD groups was significantly different from the weights of the NF group rats. However, the HFA group rats showed a slight loss compared to that reported for the HF group rats. Although intake was significantly different in the HFD groups, the HFA and HF groups were similar. In addition, the FER did not differ significantly between the HF and HFA groups throughout the experimental period.

**Table 1:** Body weight, weight gain, intake and FER of rats

	NF	HF	HFA
Initial B.W., g	272.6 ± 3.3^1NS2^	274.6 ± 2.8	269.0 ± 2.1
Final B.W.(at 16 weeks), g	356.1 ± 19.6^b^	436.5 ± 16.2^a^	419.7 ± 12.0^a^
Weight gain(for 16 weeks), g	96.4 ± 13.9^b^	161.8 ± 16.9^a^	150.8 ± 10.5^a^
Intake(for 16 weeks), g/d	17.9 ± 0.6^b^	21.2 ± 0.5^a^	19.8 ± 0.6^a^
FER^3^(for 16 weeks)	0.05 ± 0.01^b^	0.07 ± 0.01^a^	0.07 ± 0.00^a^

﻿1 Data are expressed as Mean ± S.E.(*n* = 9, NF & HF or 10, HFA)

﻿2 ﻿Values with different alphabet within the same raw are significantly different at *p* < 0.05 by Duncan’s multiple range test. NS, not significantly different

3 FER: Food efficiency ratio = weight gain(g/day)/food intake(g/day)

*Abbreviations*: *NF* normal fat diet, *HF* high fat diet, *HFA* high fat diet with 0.1% *Agrimonia pilosa* water ext

Table 2 outlines liver and epididymal fat tissue weight in the rats. The HF group rats showed significant increases in liver and epididymal adipose weights compared to that reported for the NF group rats. However, supplementation of *A. pilosa* aqueous extract decreased liver weight significantly. Although there was no statistical significance, the HFA group rats showed a tendency of reduced epididymal adipose weight compared to that reported for the HF group rats.

**Table 2:** The liver and epididymal fat tissue weight of rats

	NF	HF	HFA
Liver, g	10.74 ± 0.64^1c2^	23.1 ± 2.04^a^	18.42 ± 0.81^b^
Epididymal fat, g	2.46 ± 0.30^b^	3.6 ± 0.20^a^	3.04 ± 0.16^ab^

1 Data are expressed as Mean ± S.E.(*n* = 9, NF & HF or 10, HFA)

2 Values with different alphabet within the same raw are significantly different at *p* < 0.05 by Duncan’s multiple range test

*Abbreviations*: *NF* normal fat diet, *HF* high fat diet, *HFA* high fat diet with 0.1% *Agrimonia pilosa* water ext

### Serum glucose, insulin, and HOMA-IR

Levels of serum glucose, insulin, and HOMA-IR are shown in Table 3. Glucose levels did not differ significantly between the experimental groups. Insulin levels increased significantly in the HF group in comparison to that in the NF group. However, *A. pilosa* supplementation decreased insulin levels in the HFA group significantly. Consequently, the HOMA-IR in the HFA group was also restored to the levels seen in the NF group.

**Table 3:** The effect of *A. pilosa* on serum glucose, insulin, and HOMA-IR

	NF	HF	HFA
Glucose, mg/dL	227.1 ± 23.8^1NS2^	215.3 ± 31.9	230.1 ± 15.0
Insulin, ng/mL	3.19 ± 1.52^a^	8.75 ± 6.55^b^	4.08 ± 2.17^a^
HOMA-IR	51.55 ± 24.56^a^	134.05 ± 100.35^b^	66.80 ± 35.53^a^

1 Data are expressed as Mean ± S.E.(*n* = 9, NF & HF or 10, HFA)

2 Values with different alphabet within the same raw are significantly different at *p* < 0.05 by Duncan’s multiple range test. NS, not significantly different

*Abbreviations*: *NF* normal fat diet, *HF* high fat diet, *HFA* high fat diet with 0.1% *A. pilosa* water ext

### OGTT findings

**Fig. 1:**
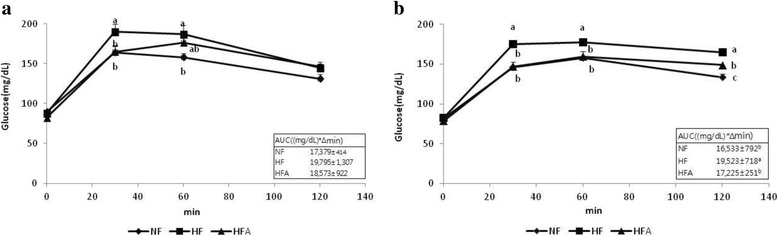
Oral glucose tolerance test at (**a**) 5 and (**b**) 15 weeks. Data are expressed as Mean ± S.E. (*n* = 9–10). Abbreviations: NF, normal-fat diet; HF, high-fat diet; HFA, high-fat diet with 0.1% *Agrimonia pilosa* aqueous extract. Values with different letters are significantly different (*p* < 0.05) by Duncan’s multiple range test

OGTT results are shown in Fig. 1. After 5 weeks of administering the experimental diets, glucose levels in the HFA group were lower than levels in the HF group 30 min after glucose administration. The AUC was not significantly different between HFD-fed groups. At 15 weeks, glucose levels in the HFA group were lower than levels in the HF group 30, 60, and 120 min after glucose administration. The AUC decreased significantly as well.

### Serum IL-6, TNF-α, and adiponectin

The effect of *A. pilosa* on serum inflammation related proteins is shown in Table 4. Levels of IL-6 and TNF-α increased significantly in the HF group in comparison to that reported for the NF group. However, the HFA group showed a significant decrease. Adiponectin levels significantly decreased in the HF group compared with levels in the NF group, but increased approximately two-fold in the HFA group compared to that in the HF group.

**Table 4:** The effect of *A. pilosa* on serum IL-6, TNF-α, and adiponectin

	NF	HF	HFA
IL-6, pg/mL	18.1 ± 8.9 ^1b2^	32.8 ± 17.5 ^a^	17.7 ± 9.8 ^b^
TNF-α, pg/mL	12.3 ± 5.8^b^	37.1 ± 21.7^a^	14.3 ± 5.1 ^b^
Adiponectin, μg/mL	153.9 ± 19.4^a^	49.6 ± 4.5^c^	96.7 ± 15.5^b^

1 Data are expressed as Mean ± S.E.(*n* = 9, NF & HF or 10, HFA)

2 Values with different alphabet within the same raw are significantly different at *p* < 0.05 by Duncan’s multiple range test

*Abbreviations*: *NF* normal fat diet, *HF* high fat diet, *HFA* high fat diet with 0.1% *A. pilosa* water ext

### Liver histology using oil red O staining

**Fig. 2:**
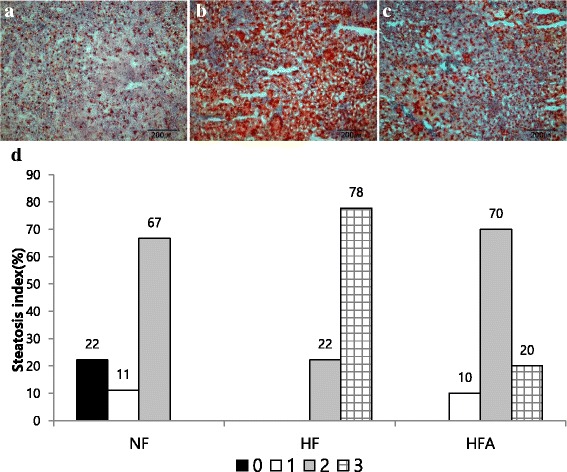
Effect of *A. pilosa* on hepatic steatosis in rats fed a high-fat diet. Photomicrographs of Oil red O-stained liver samples from representative animals in the NF (**a**), HF (**b**), and HFA (**c**) groups (× 400). (**d**) Percentage of hepatic steatosis grade (0–3 scale) within each group (*n* = 9–10). Data are expressed as percentage within group. Hepatic steatosis was graded as 0 (fatty hepatocytes occupying < 5%), 1 (fatty hepatocytes occupying 5–33%), 2 (fatty hepatocytes occupying 34–66%), or 3 (fatty hepatocytes occupying > 66%) Abbreviations: NF, normal-fat diet; HF, high-fat diet; HFA, high-fat diet with 0.1% *Agrimonia pilosa* aqueous extract

We investigated the suppressive effect of *A. pilosa* aqueous extract on lipid accumulation. Liver sections from the NF group showed a normal architecture (Fig. 2a). However, liver sections from the HF group showed a marked rise in staining within the cytoplasm of hepatocytes, indicating lipid accumulation (Fig. 2b). Lipid accumulation was reduced by *A. pilosa* treatment (Fig. 2c). The steatosis grade scores were lower in the HFA group compared to the HF group (Fig. 2d).

### Real-time quantitative reverse transcription PCR

**Fig. 3:**
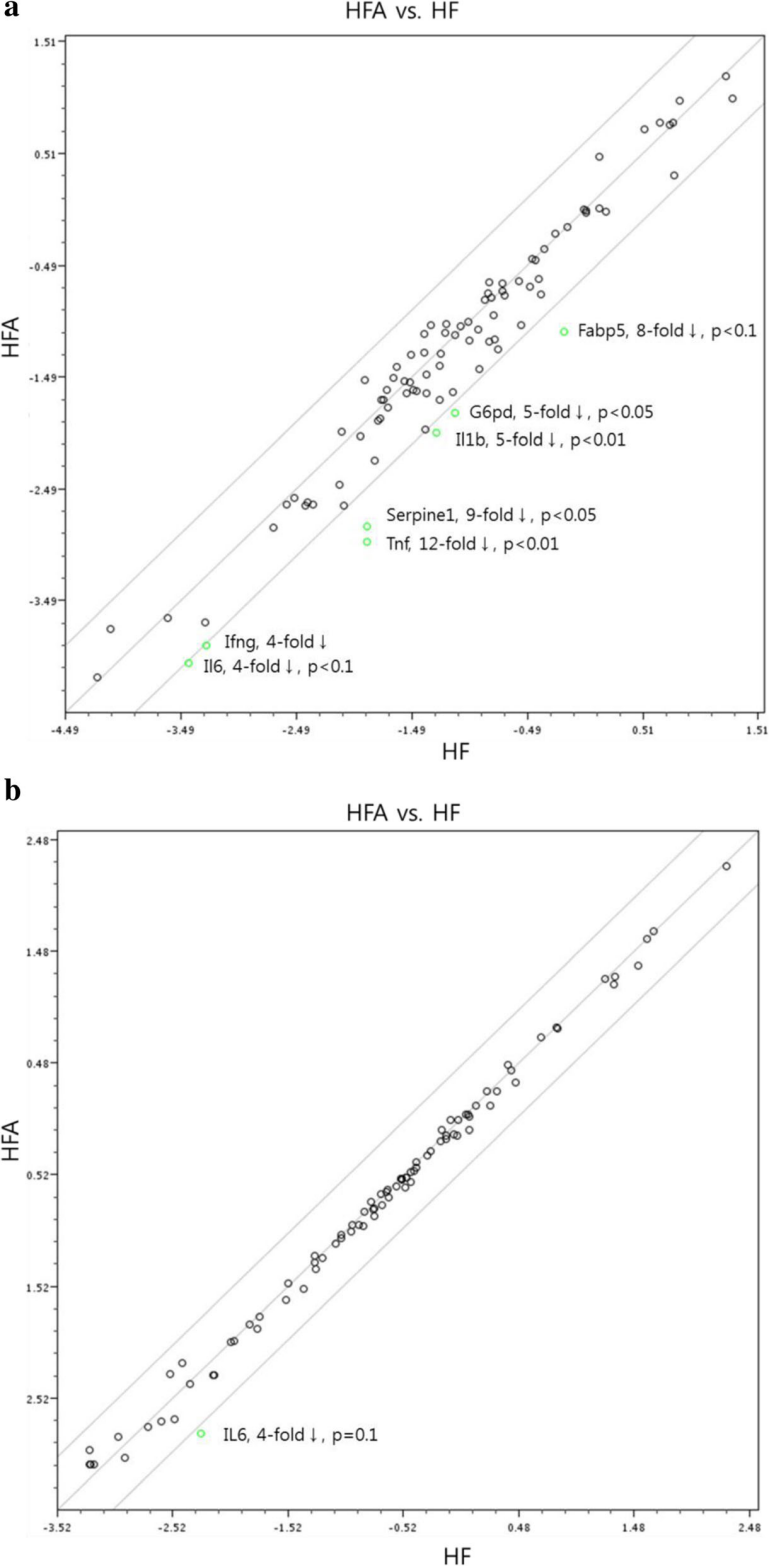
Focused quantitative RT-PCR analysis of hepatic (**a**) and adipose (**b**) tissues (*n* = 4). Abbreviations: NF, normal-fat diet; HF, high-fat diet; HFA, high-fat diet with 0.1% *Agrimonia pilosa* aqueous extract. The scatter plot compares the normalised expression of every gene on the array between two groups by plotting them against one another to quickly visualize large gene expression changes. The central *line* indicates unchanged gene expression. Data were normalised using lactate dehydrogenase A as an endogenous control

To identify inflammatory genes modulated by *A. pilosa*, we compared the hepatic gene expression profiles in the HF and HFA groups using the Rat Fatty Liver PCR array (SABiosciences). Quantitative RT-PCR array experiments showed that expression of most genes in the HFA group remained unchanged, by at least a 4-fold margin (Fig. 3a). However, *A. pilosa* treatment modulated several genes associated with insulin resistance and inflammation. Expression of the genes glucose-6-phosphate dehydrogenase (*G6pd*), interleukin 1 beta (*Il1b*), serpin peptidase inhibitor, clade E (nexin, plasminogen activator inhibitor type 1 [*PAI-1*]), member 1 (*Serpine1*), and tumor necrosis factor (TNF superfamily, member 2) (*Tnf*) was markedly downregulated in the HFA group. Although not statistically significant, Fatty acid binding protein 5 (*Fabp5)* and Interleukin 6 (*Il6)* expression decreased eight-fold and four-fold, respectively, after *A. pilosa* treatment, compared with their expression in the HF group (*p* = 0.10). Suppressor of cytokine signalling 3 (*Socs3*) was also downregulated three-fold in the HFA group.

Expression profiles of genes in epididymal tissue of the HF and HFA groups were investigated using the Rat Insulin Resistance PCR array (SABiosciences). Expression of the gene encoding IL-6 was significantly downregulated in the HFA group (Fig. 3b).

## Discussion

The present study was performed to investigate the effects of aqueous extract from *A. pilosa* on impaired glucose metabolism induced by HFD in rats. In the present study, the HF group rats had significantly higher body and liver weight, hepatic steatosis, impaired glucose tolerance, and increased insulin resistance compared to that reported for the NF group rats (*p* < 0.05). Inflammation in hepatic and adipose tissues was also induced by the HFD.

Excess calorie intake increases body weight and adipose tissue weight. Nutrient excess causes adipocyte signalling that results in inflammatory responses [17]. In mice, obese adipose tissue secretes inflammatory cytokines, TNF-α, and IL-6 [18]. In obese or HFD-fed mice, the macrophage population also increases in adipose tissues compared to that in lean controls, leading to increased cytokine expression [19]. These obesity-induced inflammatory responses may affect not only adipose tissue but important target organs, such as the liver, as well.

Obesity or HFD increases free fatty acid supply to the liver, resulting in a fatty liver and leading to hepatic inflammation through NF-κB activation and cytokine production [20]. Obese liver tissue shows an increased inflammatory cytokine expression. Although the liver is not infiltrated by macrophages in obesity-induced inflammation, hepatic inflammation is activated in macrophage-like Kupffer cells within the liver [21].

These inflammatory signals in adipose and hepatic tissues can activate c-jun N-terminal kinase (JNK) and inhibitor of κ kinase (IKK). These kinases may target insulin receptor substrate 1 (IRS-1) for serine phosphorylation, leading to inhibition of the insulin receptor signalling cascade. In the present study, the HF group showed higher adipose and liver weight, inflammation, and insulin resistance, which was confirmed by the OGTT and HOMA-IR.

However, supplementation with *A. pilosa* aqueous extract significantly decreased liver weight and serum inflammatory cytokine (TNF-α and IL-6) levels, while increasing the levels of the anti-inflammatory cytokine adiponectin. *A. pilosa* aqueous extract also reduced the expression of hepatic insulin resistance-related genes and hepatic and adipose tissue inflammation-related genes. These effects improved glucose tolerance and lowered insulin resistance in the HFA group (*p* < 0.05 vs. the HF group).

Adiponectin is an anti-inflammatory adipocytokine produced in adipose tissue. Kim et al. demonstrated that chronic overexpression of adipose tissue adiponectin decreased circulating IL-6 and TNF-α levels and reduced hepatic fat content, ameliorating insulin resistance [22]. Therefore, production of adiponectin may play a role in preventing local and systemic inflammation [23].

In our quantitative RT-PCR array experiments, expression of hepatic *G6PD*, which has been reported to be associated with insulin resistance [24], markedly decreased in the HFA group. *Serpine1* encodes PAI-1, and elevated serum PAI-1 levels are strongly correlated with insulin resistance and liver steatosis [25]. As PAI-1 expression is regulated by pro-inflammatory cytokines, this is in accordance with our findings, where *Il1b* and *Tnf* expression was markedly downregulated in the HFA group. *Socs3* expression was also downregulated in the HFA group, although not to a statistically significant extent. *Socs3* has been reported to be highly expressed in the obese liver, and to bind to the insulin receptor, inducing insulin resistance [26]. Collino et al. [27] reported that pioglitazone administration reduced hepatic inflammatory responses in rats fed a high-cholesterol fructose diet for 15 weeks, and that reduced hepatic *Socs3* expression was linked to a significant improvement in insulin resistance. Expression of the gene encoding IL-6 significantly decreased in the epididymal tissue of the HFA group. These results indicate that *A. pilosa* protected against HFD-induced insulin resistance by reducing the inflammatory response.

*A. pilosa* has been reported to exert nitric oxide-scavenging effects in lipopolysaccharide-stimulated RAW264.7 macrophages [13]. Although few previous reports have characterised the chemical components of *A. pilosa*, polyphenolic components such as flavonoids have been found [12, 28, 29]. In our previous study, flavonoids found in *A. pilosa* extract include apigenin glucuronide (21.81%), apigenin hexose (19.46%), and luteolin glucuronide (13.03%) [30]. The different molecular targets of dietary polyphenols, including apigenin and luteolin, and their anti-inflammatory effects have been well-reviewed [31]. It is expected that the positive effects of *A. pilosa* supplementation on insulin resistance can likely be attributed to these flavonoids, such as apigenin and luteolin, within *A. pilosa*. Further investigations to isolate and characterize the single compound of *A. pilosa* are needed.

These results suggest that *A. pilosa* is a healthful food for human that may potentially be used to treat obesity-related insulin resistance and metabolic syndrome. Although *A. pilosa* is edible wild plant, there is an urgent need for research to investigate the impact on human health due to the metabolic and cellular differences between rats and humans and difficulties to accurately extrapolate animal’s results to humans.

## Conclusions

The results of the present study indicate that *A. pilosa* prevents inflammatory signaling and may ameliorate impaired glucose tolerance in HFD-fed rats. *A. pilosa* may potentially be used to treat obesity-related insulin resistance and metabolic syndrome.

## Additional file

Additional file 1: Table S1. Composition of experimental diets (g/kg diet). (DOCX 15 kb)

## Abbreviations

AUC: Area under the curve
HF: High-fat diet-fed group
HFA: High-fat and *Agrimonia pilosa* aqueous extract-fed group
HFD: High-fat diet
HOMA-IR: Homeostasis model assessment of insulin resistance
IL: Interleukin
NAFLD: Nonalcoholic fatty liver disease
NASH: Nonalcoholic steatohepatitis
NF: Normal-fat diet-fed group
OGTT: Oral glucose tolerance test
PAI-1: Plasminogen activator inhibitor type 1
TNF: Tumor necrosis factor
