# Supplementary material for: Agrimonia pilosa Ledeb. aqueous extract improves impaired glucose tolerance in high-fat diet-fed rats by decreasing the inflammatory response
Source: BMC Complement Altern Med. 2017 Sep 5;17:442. doi: 10.1186/s12906-017-1949-z (PMC5583762; doi:10.1186/s12906-017-1949-z)
Supplement: Additional file 1: Table S1. — Composition of experimental diets (g/kg diet). (DOCX 15 kb) [file 12906_2017_1949_MOESM1_ESM.docx]

**Table 1. Composition of experimental diets (g/kg diet)**

|  | NF | HF | HFA |
| --- | --- | --- | --- |
| Cornstarch | 465.692 | 315.192 | 314.192 |
| Casein | 140 | 140 | 140 |
| Dextrinized cornstarch | 155 | 155 | 155 |
| Sucrose | 100 | 100 | 100 |
| Beef tallow | 40 | 180 | 180 |
| Cholesterol | - | 10 | 10 |
| Fiber | 50 | 50 | 50 |
| Mineral mixture^2^ | 35 | 35 | 35 |
| Vitamin mixture^3^ | 10 | 10 | 10 |
| L-cystein | 1.8 | 1.8 | 1.8 |
| Choline bitartrate | 2.5 | 2.5 | 2.5 |
| TBHQ^4^ | 0.008 | 0.008 | 0.008 |
| Cholic acid |  | 0.5 | 0.5 |
| *Agrimonia pilosa* water ext. | - | - | 1 |

1 Abbreviations: NF, normal fat diet; HF, high fat diet; HFA, high fat diet with 0.1% *Agrimonia pilosa* water ext.

2 Mineral mixture : AIN-93M mineral mixture(ICN, CA, USA)

3 Vitamin mixture : AIN-93VX vitamin mixture(ICN, CA, USA)

4 TBHQ : Tert-butylhydroquinone
